# Supplementary material for: Resolving the full spectrum of human genome variation using Linked-Reads
Source: Genome Res. 2019 Apr;29(4):635–45. doi: 10.1101/gr.234443.118 (PMC6442396; doi:10.1101/gr.234443.118)
Supplement: Supplemental Material [file supp_gr.234443.118_Supplemental_longranger-2.2.zip › longranger-2.2.tar.bz2!/lr2.2-public/lib/stan/cmdstan/stan/doc/api/html/classstan_1_1agrad_1_1op__vvv__vari-members.html]

Stan: Member List


|  |  |
| --- | --- |
|  | Stan  2.5.0  probability, sampling & optimization |


- Main Page
- Related Pages
- Namespaces
- Classes
- Files

- Class List
- Class Index
- Class Hierarchy
- Class Members

All Classes Namespaces Files Functions Variables Typedefs Enumerations Enumerator Friends Macros Pages

- stan
- agrad
- op\_vvv\_vari

stan::agrad::op\_vvv\_vari Member List

This is the complete list of members for stan::agrad::op\_vvv\_vari, including all inherited members.

|  |  |  |
| --- | --- | --- |
| adj\_ | stan::agrad::vari |  |
| avi\_ | stan::agrad::op\_vvv\_vari | protected |
| bvi\_ | stan::agrad::op\_vvv\_vari | protected |
| chain() | stan::agrad::chainable | inlinevirtual |
| chainable() | stan::agrad::chainable | inline |
| cvi\_ | stan::agrad::op\_vvv\_vari | protected |
| init\_dependent() | stan::agrad::vari | inlinevirtual |
| op\_vvv\_vari(double f, vari \*avi, vari \*bvi, vari \*cvi) | stan::agrad::op\_vvv\_vari | inline |
| operator delete(void \*) | stan::agrad::chainable | inlinestatic |
| operator new(size\_t nbytes) | stan::agrad::chainable | inlinestatic |
| set\_zero\_adjoint() | stan::agrad::vari | inlinevirtual |
| val\_ | stan::agrad::vari |  |
| vari(const double x) | stan::agrad::vari | inline |
| vari(const double x, bool stacked) | stan::agrad::vari | inline |
| ~chainable() | stan::agrad::chainable | inlinevirtual |
| ~vari() | stan::agrad::vari | inlinevirtual |


---

[ Stan Home Page ]

*© 2011–2014,
Stan Development Team.*
